# Clustering-based approaches to SAGE data mining

**DOI:** 10.1186/1756-0381-1-5

**Published:** 2008-07-17

**Authors:** Haiying Wang, Huiru Zheng, Francisco Azuaje

**Affiliations:** 1 School of Computing and Mathematics, University of Ulster, Newtownabbey, BT37 0QB, Co. Antrim, Northern Ireland, UK; 2 Research Centre for Public Health (CRP-Santé), Laboratory of Cardiovascular Research, 1A, rue Thomas Edison, L-1445, Strassen, Luxembourg

## Abstract

Serial analysis of gene expression (SAGE) is one of the most powerful tools for global gene expression profiling. It has led to several biological discoveries and biomedical applications, such as the prediction of new gene functions and the identification of biomarkers in human cancer research. Clustering techniques have become fundamental approaches in these applications. This paper reviews relevant clustering techniques specifically designed for this type of data. It places an emphasis on current limitations and opportunities in this area for supporting biologically-meaningful data mining and visualisation.

## Background

Serial analysis of gene expression (SAGE) [1] is one of the most powerful, high-throughput tools available for global gene expression profiling at mRNA level. It allows quantitative, simultaneous analysis of thousands of transcript profile in a cell or tissue under specific biological conditions without requiring prior, complete functional knowledge of the genes to be analysed. Basically, the technique of SAGE relies upon on two fundamental principles [2]: (1) a short nucleotide sequence, or *SAGE tag*, isolated from a defined position within an mRNA transcript is assumed to include sufficient information to uniquely represent the transcript and; (2) the end-to-end concatenation of tags into long DNA molecular allows rapid, efficient sequencing analysis of multiple transcripts. It has been shown that SAGE has several advantages over other gene expression analysis techniques. For example, unlike RNA blotting and RT-PCR, SAGE is able to examine thousands of transcript profiling at the same time. Unlike DNA microarray technologies, which is limited to the analysis of genes previously characterised and assigned to an array, the SAGE approach allows for the detailed analysis of expression patterns of uncharacterised genes as well as of known genes. Furthermore, the output of SAGE-based analysis is the digital measurement of absolute RNA abundance levels, greatly facilitating direct and reliable comparison of expression profiles produced by different experiments and laboratories [3,4]. Such unique features have led to many important applications in a wide variety of studies, such as the discovery of potential transcriptional regulators and construction of biological networks [5], the identification of novel molecular tumour markers and therapeutic targets [6], the study of the molecular profile of gastroesophageal junction carcinomas [7] and the genomic analysis of mouse retinal development [8]. Computational pattern discovery and classification based on data clustering plays an important role in these applications. However, due to the unique characteristics of SAGE data, mining this type of data poses a great challenge to the bio-data mining community.

### Characteristics of SAGE data

As a result of global expression profiling, SAGE data are characterised by the presence of large amounts of high-dimensional data and the absence of functional and structural knowledge of many derived tags. For example, the result of a SAGE experiment, known as a *SAGE library*, is usually composed of many thousands of sequenced tags. Each tag is associated with a discrete value representing the expression level of a transcript in a particular tissue sample under selected physiological conditions. Furthermore, the generation of SAGE data does not rely on known gene sequence information. As a consequence, biological functions of many derived SAGE tags may remain largely unknown. For instance, in a study conducted by El-Meanawy *et al*. [9], a SAGE library generated from mouse kidney was constructed. It consisted of 3,868 sequence tags, in which only 42 percent of the transcripts matched mRNA sequence entries with known functions. Biological functions of 58 % of the transcripts were unknown.

Raw SAGE data may come in a variety of noisy representations and may include artifact sequences resulting from various sources of errors that are inherent in the experimental processes involved in the generation of data [10]. It is estimated that the number of tags containing artificial counts caused by sequencing errors counts to be between 5% and 15% of the total number of tags [11,12]. A study carried out by Akmaev and Wang [10] suggested that 3.5% of 21 bp (base pairs) SAGE tag, generated by a enhanced SAGE protocol (LongSAGE) [13], tags have errors inherited from the polymerise chain reaction (PCR) amplification and 17.3% of the tags in LongSAGE libraries have errors resulting from sequencing errors. It has been shown that the occurrences of these errors could lead to significant biases in the observed results produced by SAGE [14]. The techniques used to remove sequence errors and correct relevant artefacts can be found in [10,11], and [15]. Due to the inherent nature of their data acquisition procedure, SAGE data are governed by different statistical models from those of array-based gene expression analysis. Let *p*(*x*) be the probability of observing *x *counts of a given tag in a specific SAGE library and λ be the expected count for a given tag, it has been suggested that the number of sampled transcripts of a particular type observed in a given SAGE library closely follows a Poisson distribution [16], i.e.

(1) *p*(*x*) = exp(-*λ*) × *λ*^*x*^/*x*!

Moreover, such distributions are independent of each other across different SAGE tags and SAGE libraries as pointed out by Cai *et al*. [17].

### SAGE data analysis: An overview

In recent years there has been an accumulation of significant amounts of SAGE data generated from different tissues and cell lines across different species such as human and mouse. However, such vast collections of data are not in themselves useful. In an attempt to extract useful knowledge encoded in the data, different data mining techniques have been developed and applied to analyze SAGE data. For example, several statistical tests have been used to study differential expression of genes based on the pairwise comparisons of SAGE libraries. Examples include Fisher's Exact test [18], Monte Carlo simulation-based test [1] and Bayesian statistics-based approaches [19]. A comparative review of these applications can be found in [20].

To identify differential expression in multiple SAGE libraries, several statistical models were proposed to account for both between-library and within-library variation. Based on a hierarchical beta-binomial model, Baggerly *et al*. [21] introduced a statistic test, *t*_*w*_, for two-group comparisons. To simultaneously model multiple types of variance and deal with multiple groups, a model based logistic regression with over-dispersion was introduced in [22]. A comparative evaluation of these approaches was conducted by Lu *et al*. [23].

Apart from these statistical test-based methods, other data mining techniques based on machine learning approaches (e.g. artificial neural networks) have also been applied to make SAGE data meaningful. Becquet *et al*. [24] utilized the association-rules discovery technique to reveal strong association rules hidden in large-scale human SAGE data. Rioult *et al*. [25] proposed an inductive database approach for mining biologically meaningful concepts from large SAGE expression data. Jin *et al*. [26] studied the performance of four supervised classification models, i.e. Support Vector Machine (SVM), Naïve Bayes (NB), Nearest Neighbour and C4.5 for cancer classification based on SAGE data, with SVM and NB achieving the best prediction performance. A Chi-square-based feature selection was used to deal with the high dimensional problem inherent in SAGE data. To support the identification of photoreceptor enriched genes based on SAGE expression data, Wang *et al*. [27] investigated three machine learning-based models (*KStar*, C4.5 decision tree, and *multilayer perceptron *neural network) for inferring functional associations from the SAGE data. Surprisingly, *KStar*, a relatively simple instance-based model performed significantly better than more complex algorithms, e.g. neural networks.

Being capable of detecting potentially novel and significant transcript or gene groups, clustering-based approaches have received great attention. For instance, based on hierarchical clustering analysis of 88 human cancer SAGE libraries, Ng *et al*. [28] presented a method to detect similarities between different types of cancer at the sub-cellular level. By modelling SAGE data with a Poisson distribution, Cai *et al*. [17] proposed a new K-means clustering technique to analyse SAGE data. More recently, Zheng *et al*. [29] introduced a novel self-adaptive neural network to supporting pattern discovery and visualization in SAGE data.

This paper places an emphasis on clustering-based approaches to SAGE data mining. Applications of traditional clustering techniques to analyse SAGE data are introduced in the next section, followed by a review of current advances in clustering analysis of SAGE data. The assessment of the quality of clustering techniques will also be investigated.

## Clustering-based approaches to SAGE data mining: traditional techniques and their applications

A typical SAGE library consists of a list of thousands of tags and the number of times each tag is observed in a particular tissue sample obtained from different physiological conditions. A SAGE dataset can be summarized by a matrix, in which each horizontal row represents a sequenced SAGE tag and vertical columns contain various SAGE libraries corresponding to either serial time points taken from different development stages of a biological process or to various biological conditions. After data preprocessing, such as removal of sequencing and sampling errors, this matrix can then be analyzed by various clustering techniques. The main objectives are to cluster tags or libraries into classes that can be differentiated on the basis of their expression patterns and to identify groups of tags (or libraries) sharing similar expression patterns. This can be achieved by a two-way cluster analysis of the matrix: (1) clustering of SAGE tags based on expression profiles of each individual tag; and (2) clustering of SAGE libraries based on the expression profiles of each library.

Similarity measure between pairs of patterns is essential in most of clustering techniques. In the context of SAGE data analysis, the popular measure is based on the calculation of Pearson correlation across different libraries (or tags). Recently, based on the consideration of statistical nature of SAGE data, several Poisson-based similarity measures have been proposed, which will be further discussed in the next section.

**Figure 1:**
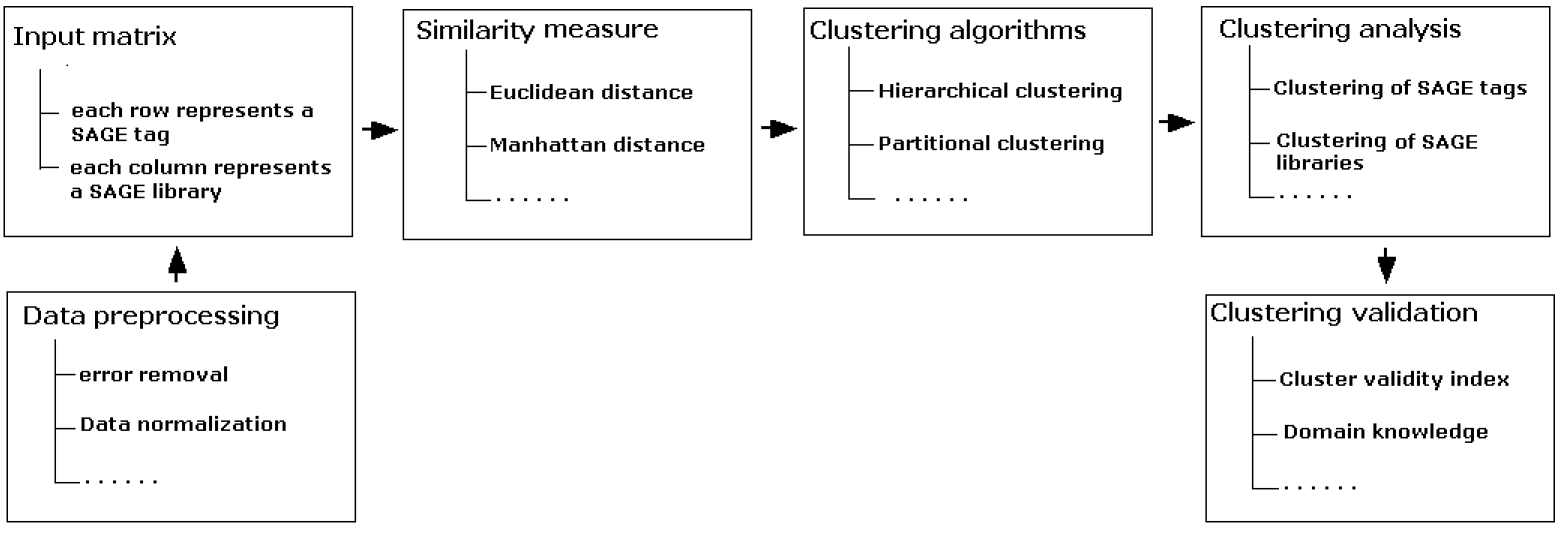
An overview of the basic steps involved in clustering-based approach to SAGE data mining.

The quality of the clustering outcomes can be assessed using different clustering validation techniques [30]. Mapping SAGE tags to kown genes [31] can also be used to support the estimate of the quality of SAGE cluster analysis. Figure 1 summarises the basic steps involved in clustering analysis of SAGE data.

Based on the observation that genes exhibiting similar expression patterns are more likely to be co-regulated and share similar biological functions [32], clustering-based SAGE data analysis has found different applications, for example, the identification of biomarkers in human cancer research [33], the discovery of cell- specific promoters modules [34], and the better understanding of transcriptional networks [35,36]. Such applications mainly rely on the following traditional clustering techniques.

### Hierarchical clustering

By being able to construct a hierarchy of clusters and diagrammatically summarise the clustering process in a tree form, i.e. dendrogram, hierarchical clustering has become one of the most widely used clustering techniques in SAGE data analysis. It can be implemented by using agglomerative and divisive approaches. Starting from each tag assigned to its own cluster, an agglomerative technique forms the cluster structure in a bottom-up fashion until all SAGE tags belong to the same cluster. Depending on the way of defining similarity between clusters, such an approach has several variations such as single linkage, average linkage and complete linkage methods. A divisive method takes a top-down approach. It begins with the whole dataset as a single cluster, and iteratively splits up existing clusters into smaller clusters until each cluster only contains one data sample. Examples of hierarchical clustering for SAGE data analysis include a recent study conducted by Lee *et al*. [5], who clustered SAGE data obtained from the transcriptome of mouse type A spermatogonia, pachytene spermatocytes, and round spermatids to support the identification and discovery of potential transcriptional regulators and pathways involved in different stages of spermatogenesis. Based on the hierarchical clustering of 88 SAGE libraries derived from cancerous and normal tissues, as well as cell line material, Sander *et al*. [37] systematically studied cancer expression profiling. They found that based on SAGE expression data, brain and breast cancer samples could be clearly discriminated from their normal counterparts, but not in the case of prostate and ovarian cancers. To identify molecular alterations involved in the initiation and progression of breast carcinomas, Porter *et al*. [38] applied hierarchical clustering to analyze eight SAGE libraries generated from normal and cancerous human breast tissues.

**Figure 2:**
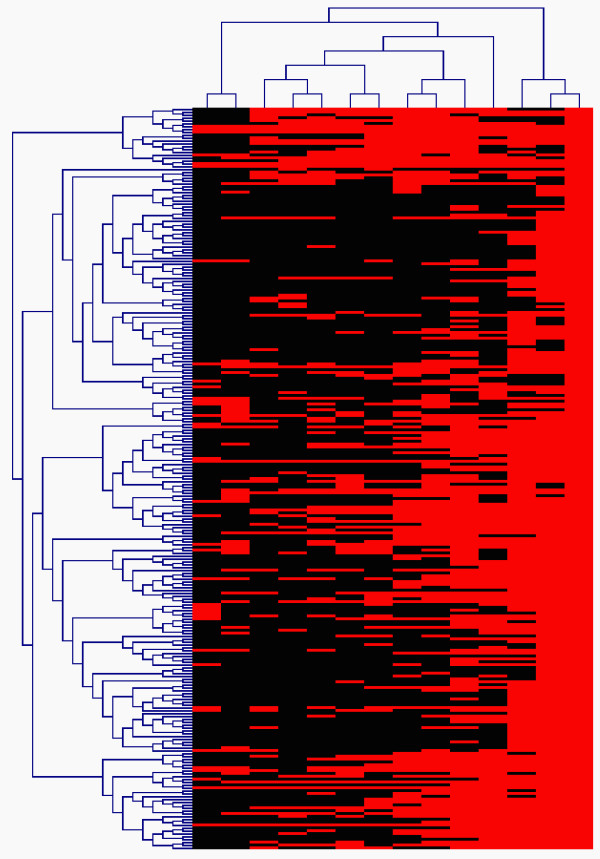
**An illustration of two-way hierarchical clustering analysis of 1118 SAGE tags highly expressed in the mouse microdissected outer nuclear layer (ONL) published by Blackshaw et al**. [39]. Each row represents a SAGE tag, where each columns correspond to a SAGE library. A total of murine 14 libraries were considered including different tissues and developmental stages, including mouse NIH-3T3 fibroblast cells, adult hypothalamus, developing retina at 2 day intervals from embryonic day (E) 12.5 to postnatal day (P) 6.5, P10.5 retinas from the paired-homeodomain gene crx knockout mouse (crx-/-) and from wild type (crx+/+) littermates, adult retina and microdissected outer nuclear layer (ONL). developing retina at 2 day intervals from embryonic day (E) 12.5 to postnatal day (P) 6.5, P10.5 retinas from the paired-homeodomain gene crx knockout mouse (crx-/-) and from wild type (crx+/+) littermates, adult retina and ONL.

The dendrogram is a graphical representation of hierarchical clustering, in which each step of the clustering process is illustrated by a tree joint, and each tree node represents a subset of expression data provides. It provides an intuitive platform for biologists to visualize basic relationships between all the tags or libraries, as illustrated in Figure 2. This example shows a two-way hierarchical clustering of 1118 SAGE tags highly expressed in the mouse microdissected outer nuclear layer (ONL) published by Blackshaw *et al*. [39]. However, such a representation does not directly produce explicit partitions of the data. Given the sheer number of the data possibly involved in the analysis of SAGE studies, it is usually not obvious how to define clusters from the tree. For example, it could be a complex task for users to determine the optimal number of clusters and obtain meaningful partitions solely based on the dendrogram shown in Figure 2.

### *K*-means clustering

The *k*-means method is perhaps one of the simplest, best known clustering techniques. It partitions a dataset into *k *clusters iteratively, such that (1) each sample is assigned to its closest centroid, and (2) the dispersion within *k *clusters is minimised. Mechaly *et al*. [35] applied *k*-means clustering to study transcriptional networks involved in the mouse adult peripheral nerve repair program. Four SAGE libraries taken from mouse dorsal root ganglia at embryonic day E13, neonatal day P0, adult and adult 3 days post-axotomy were analysed. A *k*-means clustering with *k *= 50 was performed on 5400 SAGE tags. This analysis led to the identification of candidate genes, such as *DDIT3*, *TIMM8B*, and *OAZIN*, as potential injury-induced molecular actors involved in a stress response pathway.

*K*-means clustering exhibits several limitations that hinder its performance. One of its fundamental disadvantages is that the output of the *k*-means procedure is an unorganised collection of clusters that is not always conductive to biological and physiological interpretation [40]. Nevertheless, the simplicity and scalability among large datasets still make *k*-means clustering technique an attractive alternative when dealing with large SAGE datasets.

### Self-organizing map (SOM)

**Figure 3:**
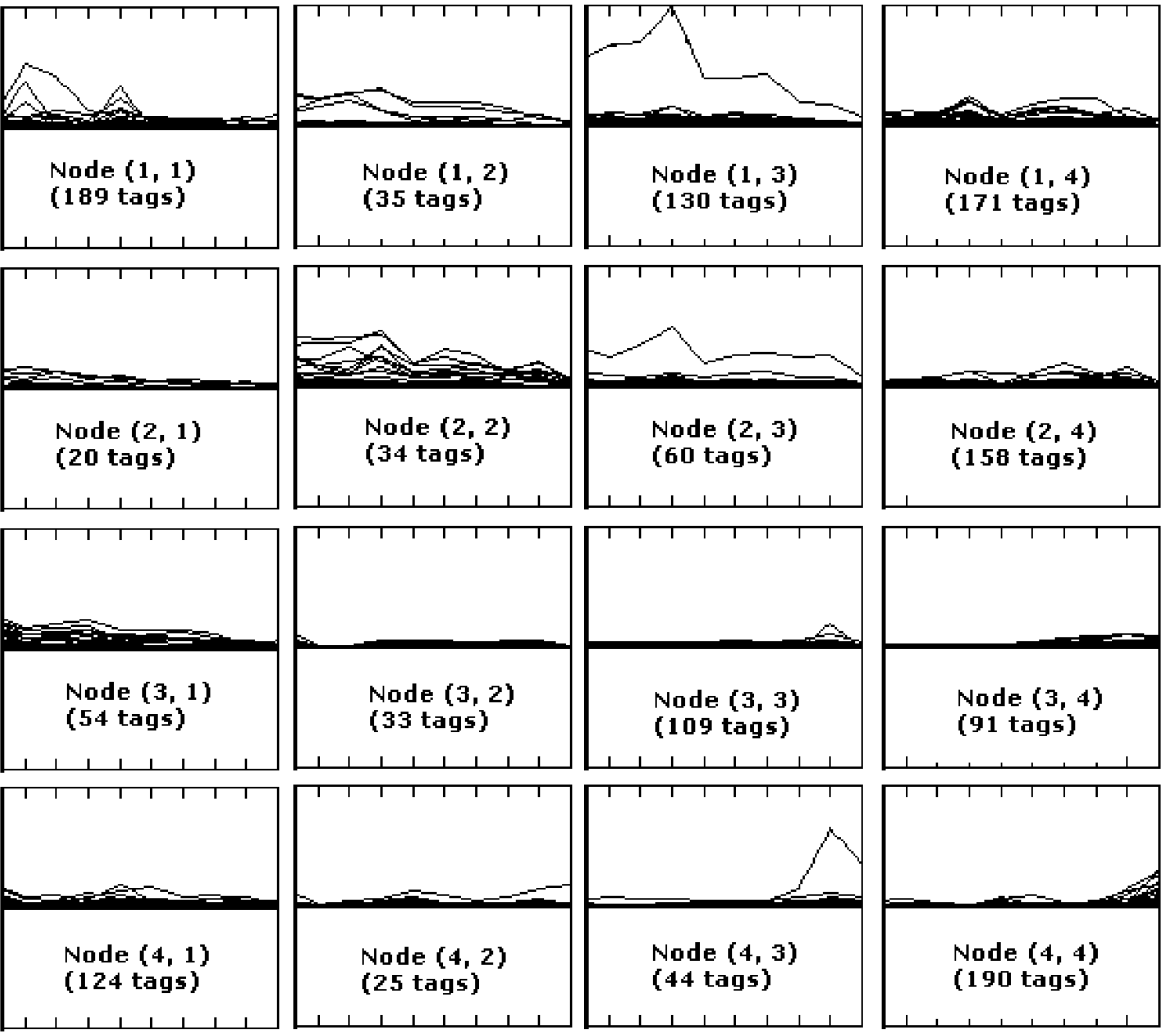
**The expression profiles of the SOM based on the analysis of 1467 SAGE tags **[23,22]. Ten SAGE libraries10 from developing mouse retina taken at 2-day intervals from embryonic day 12.5 (E12.5) to postnatal day 10.5 (P10.5) and adult retina are plotted on the x-axis, and relative tag abundance is shown on the y-axis. The number shown on each graph stands for the total number of tags in each cluster.

The basic idea of the SOM [41] is to produce a low dimensional (usually a 2- dimensional grid) representation of a high dimensional input space while preserving key similarity relations between input data samples. The resulting map is characterised by the formation of a topological map of the original data, in which similar patterns (i.e. samples) are close to each other, and the ones that are less similar tend to be further away. Figure 3 shows a 4 × 4 SOM map based on the analysis of 1467 SAGE tags published by [22]. Ten SAGE libraries from developing mouse retina taken at 2-day intervals from embryonic day 12.5 (E12.5) to postnatal day 10.5 (P10.5) and adult retina were plotted on the x-axis, and relative tag abundance is shown on the y-axis of each SOM node. It can be seen that most of the tags, which show higher expression level in embryonic day are grouped together on the top-left hand-side in Figure 3, while those SAGE tags exhibiting higher expression values during postnatal periods are clustered into the right-bottom nodes. The tags with varying expression patterns throughout retinal development tend to be allocated in the central nodes shown in Figure 3.

The ability to reveal the intrinsic cluster structure of the data in a low dimensional space makes the SOM an appealing and powerful tool in various forms of clustering analysis. In a recent study published by Mclntosh *et al*. [42], the SOM has successfully supported the study of gene expression profiling of developing wheat caryopsis. A total of five SAGE libraries were constructed at five post-anthesis time-points, which correlate to key stages in the caryopsis developmental process. More than 90,000 LongSAGE tags were sequenced generating 29,261 unique tag sequences across all five SAGE libraries. Based on clustering analysis of expression patterns of the 250 most abundant tags with SOM, differential expression profiles that highlight development-specific genes were identified.

The application of the SOM-based clustering techniques requires the network structure and the number of nodes to be specified by the user in advance. Currently, for an unknown dataset, there is no theoretical way to determine the optimal size and structure of the output network. Usually, users need to rely on a trial-and-error method, which undoubtedly represents a time-consuming and tedious task.

## Current advances in clustering analysis of SAGE data

### Clustering analysis with a Poisson approach

Most of the applications discussed in the last section mainly applied tools currently available in microarray data clustering tools such as TIGR MeV [35] and GeneSpring [42]. The data clustering algorithms offered by these tools do not take into account the specific statistical nature inherent in SAGE data. It has been shown that without the incorporation of the statistical model exhibited by SAGE data into the learning process, the advantages of clustering-based approaches may not be fully realized [17].

Based on the assumption that the number of tags observed in a SAGE library closely follows a Poisson distribution, Cai *et al*. proposed a new clustering algorithm (PoissonC) specifically designed for SAGE data. It was implemented within the *k*-means clustering framework but with a Poisson statistics-based function as a similarity measure. One of the innovations of PoissonC was that, instead of using traditional distance measures such as Pearson correlation and Euclidean distance, it adopts the chi-square statistic to determine how to assign each SAGE tag to its closest (cluster) centroid. Tested on simulated and experimental mouse retinal SAGE data, PoissonC has demonstrated significant advantages over traditional clustering methods.

More recently, Wang and colleagues [43] further incorporated Poisson statistics-based similarity measures into the learning process of SOM and agglomerative hierarchical clustering algorithms. Two new clustering methods called PoissonS and PoissonHC respectively were proposed. Like in PoissonC, PoissonS utilized Chi-square statistics to determine the winning nodes for each input sample. Let *Y*_*i *_be the input vector representing the *i*^*th *^*k*-dimensional SAGE tag (*k *is the total number of SAGE libraries), ${\hat{Y}}_{i}(t)$ be the expected value of *Y*_*i*_(*t*) (*t *is the index of SAGE library), and *m*_*j *_be the associated weight vector of the *j*^*th *^node, PoissonS used the following minimum Chi-square statistics-based distance matching criterion to determine the winning node denoted by the subscript *c*:

(2) dχ(i,j)=∑t=1k((Yi(t)−Yˆ(t)i)2Yˆi(t))

(3) $${\hat{Y}}_{i}(t)=(m_{j}(t)/\sum_{t=1}^{k}\left(m_{j}(t)\right))\times \sum_{t=1}^{k}Y_{i}(t)$$

(4) *d*_*χ*_(*i*, *c*) = min *d*_*χ*_(*i*, *j*),   ∀ *j*

The calculation of the expected count represented in Equation (3) considers the following factors: (a) Like in a SOM, after each learning epoch, the weight vector, *m*_*j*_, in the PoissonS coincides with the centroid of the respective cluster, and (2) the main purpose of PoissonS is to group tags with similar relative expression rather than the absolute expression levels.

Under the assumption that Poisson distributions governing the generation of SAGE data are independent of each other across different SAGE tags and libraries, PoissonHC used the joint likelihood function, *p*(*i*,*j*), as a distance function to measure the similarity between tags *i*^*th *^and *j*^*th *^tags.

(5) $$p(i,j)=\prod_{t=1}^{k}(\exp(-{\hat{Y}}_{i}(t)){\hat{Y}}_{i}{\left(t\right)}^{Y_{i}(t)}/Y_{i}(t)!)\times \prod_{t=1}^{k}(\exp(-{\hat{Y}}_{j}(t)){\hat{Y}}_{j}{\left(t\right)}^{Y_{j}(t)}/Y_{j}(t)!)$$

Where ${\hat{Y}}_{i}(t)$ and ${\hat{Y}}_{i}(t)$ are the expected values of *Y*_*i*_(*t*) and *Y*_*j*_(*t*) respectively, which can be calculated by using the 2 × *k *contingency table with *Y*_*i*_(*t*) being the first row and *Y*_*j*_(*t*) being the second row [44].

**Figure 4:**
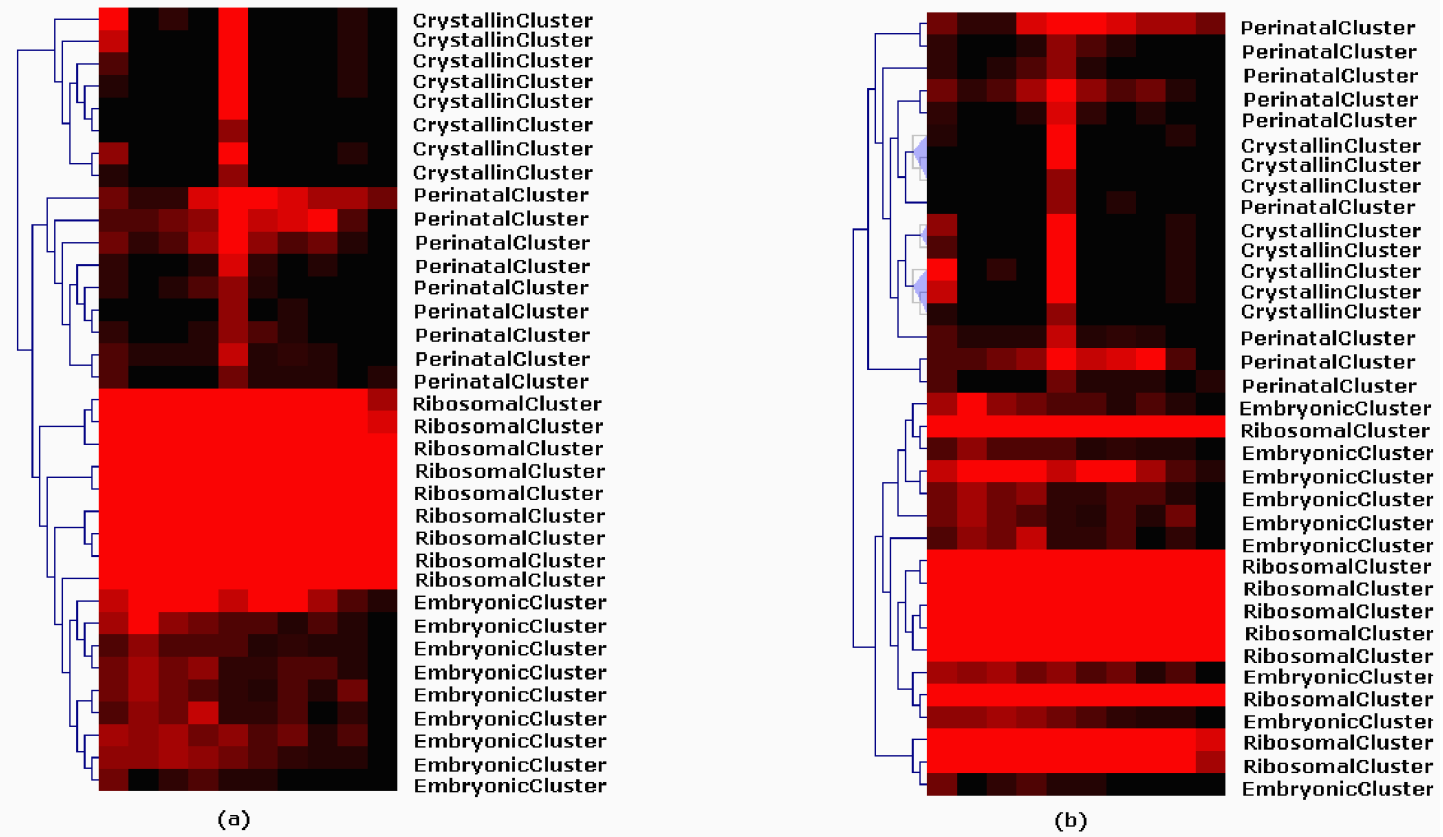
**Hierarchical cluster analysis on the 35 tags with known biological functions and distinctive expression patterns with (a) PoissonHC and (b) hierarchical clustering with Pearson correlation as a distance function (adapted from **[43]). The 35 SAGE tags under consideration includes 8 crystallin proteins (*CrystallinCluster*), 9 tags whose expression peak appears around P0.5 (*PerinatalCluster*), 9 ribosomal proteins (*RibosomalCluster*), and 9 tags whose peak expression pattern occurs before E16.5 (*EmbryonicCluster*).

The performance of both PoissonS and PoissonHC was evaluated by using three datasets, i.e. one synthetic set published by Cai *et al*. [17], mouse retinal SAGE data including 10 murine SAGE libraries generated from developing retina taken at 2-day intervals [8] and human cancer SAGE data including eleven human cancer SAGE libraries [45]. The results indicated that, in the context of SAGE-based data clustering, both PoissonS and PoissonHC offer several advantages over existing traditional data clustering techniques techniques. Figure 4 shows clustering analysis of a set of 35 tags with known biological functions and distinctive expression patterns with PoissonHC and hierarchical clustering with Pearson correlation as a distance function. Clearly, PoissonHC outperformed its hierarchical clustering counterpart.

**Figure 5:**
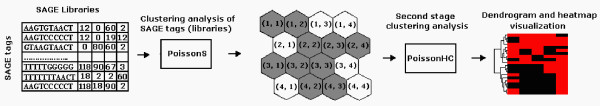
An example of the combination of PoissonS and PoissonHC for SAGE data analysis with PoissonS as the first analysis level and Poisson HC clustering prototypes originating from PoissonS and SAGE tags assigned to each node. Such a combination may highlight inter- and intra-cluster relationship hidden in the SAGE data.

To further enhance the capability for pattern discovery and visualization in SAGE data, a hybrid approach based on the combination of PoissonS and PoissonHC with PoissonS as the first analysis level, as illustrated in Figure 5, was also proposed by Wang *et al*. [43]. Such a combination allows a better understanding of inter- and intra-cluster relationships hidden in the SAGE data.

### Self-adaptive neural networks (SANNs)

SANNs represent a family of unsupervised neural networks, which have ability of dynamically organizing themselves (i.e. automatically adapt its topology) according to the natural clustering structure of the underlying data. Unlike the SOM, whose topology and number of nodes need to be predetermined by the user, SANNs allow the structure as well as the size of the network to be determined during the learning process. Thus, the resulting map has a structure that is directly linked to the underlying dataset. From a clustering prospective, such a feature may greatly facilitate the identification of cluster structures hidden in the data.

**Figure 6:**
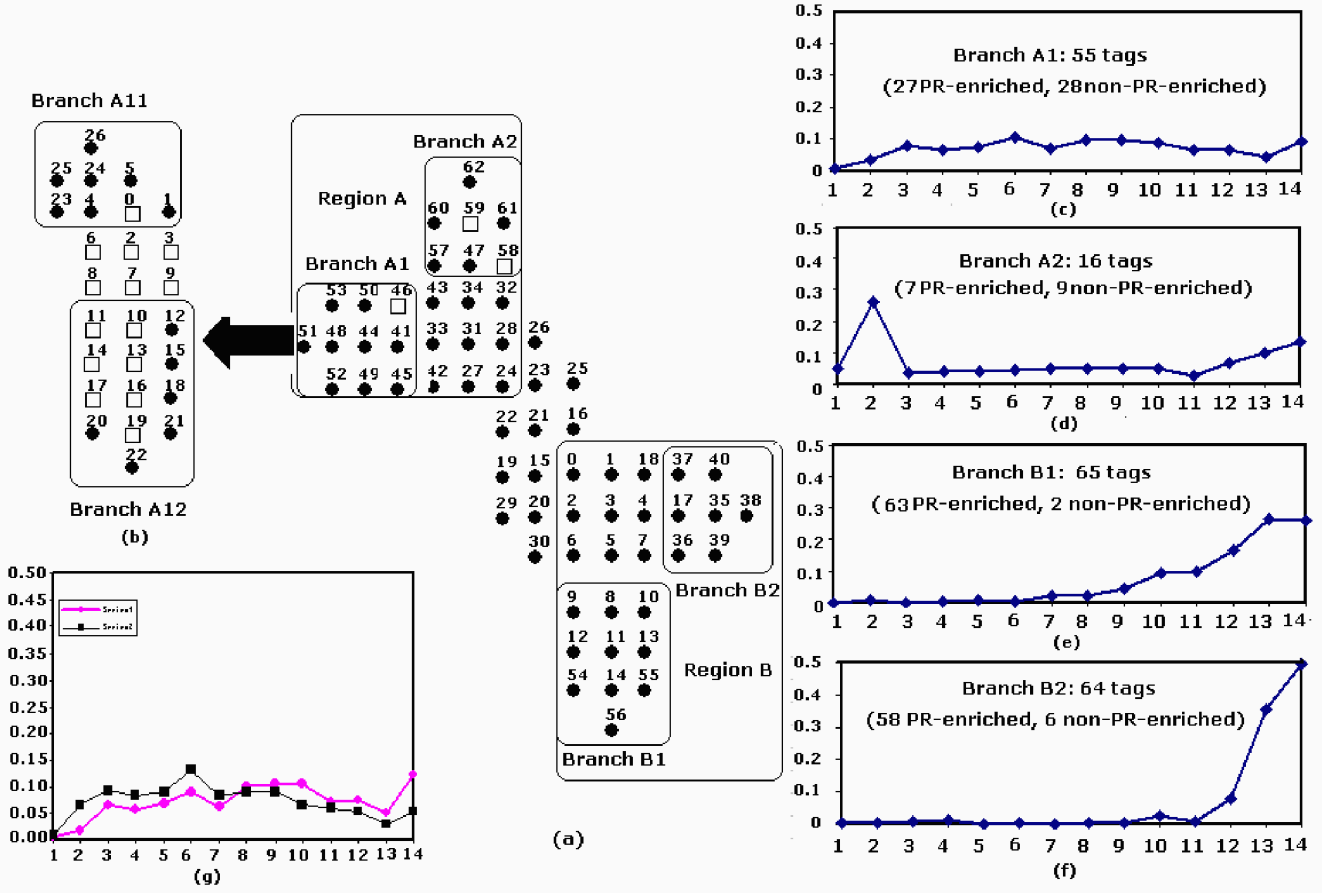
**PGSOM-based data analysis for mouse retinal SAGE data. (a) A representative output map. (b) The submap is a higher-resolution map for Branch A1**. (c) to (g) The median plots of expression patterns represented by Branches A1, A2, B1, B2, A11 and A12 respectively. Ten SAGE libraries are plotted on the x-axis, and relative expression abundance over 14 libraries is shown on the y-axis. The numbers shown on the x-axis represent 14 SAGE libraries, i.e., 1→3t3; 2→hypo; 3→E12.5; 4→E14.5; 5→E16.5; 5→E18.5; 7→P0.5; 8→P2.5; 9→P4.5; 10→P6.5; 11→P10.5Crx-/-; 12→P10.5Crx+/+; 13→Adult; 14→ONL. The total numbers of tags that fall into each branch along with the distribution of PR-enriched and non-PR-enriched tags over branches are shown on graphs (c) to (f).

Zheng *et al*. [29] recently reported a new SANN model, *Poisson-based Growing Self-Organizing Map *(PGSOM), which implements novel weight adaptation and neurone growing strategies by taking into account the statistical properties of SAGE data. A fundamental advantage of PGSOM is that, based on the implementation of a Poisson statistic-based topology adaptation strategy, it is able to reflect similarity relationships and expression patterns encoded in the SAGE data by branching out. Figure 6 shows a representative map of PGSOM based on the analysis of a mouse retinal SAGE dataset published by Blackshaw *et al*. [39], which includes 63 non-PR-enriched and 261 PR-enriched tags. As can be seen from Figure 6(a), the PGSOM resulting map has branched out into four directions (Branches A1, A2, B1, and B2), each representing a distinct expression pattern encoded in the SAGE data. For example, genes associated with tags in Branches B1 are not associated with non-retina tissue (3t3 and hypo libraries) and before postnatal day P6.5. However, a significant increase in expression was observed throughout postnatal day. Genes that fall into Branch A12 show comparatively early onset of expression with expression signature starting at early stage of embryonic day and peaking around the time of birth.

Interestingly, PGSOM can also be used to perform hierarchical and multi-resolution clustering on selected areas of interest based on the selection on different learning parameters. The submap shown in Figure(b) is a higher-resolution map of Branch A1. This branch expanded into two directions: Branches A11 and A12, which were separated by the area covered by the dummy nodes. An analysis of expression pattern of SAGE tags clustered into these sub-branches reveal that each sub-branch is associated with a distinct, prototypical expression profile. While tags found in sub-branch A11 typically demonstrate a peak in expression during embryonic day, sub-branch A12 mainly contains tags with expression levels peaking around postnatal day, as shown in Figure 6(g).

### Semi-supervised clustering approach

Traditional data-driven clustering analysis of SAGE data ignores existing prior biological knowledge. Recent advances in clustering techniques have demonstrated how functional biological knowledge can be integrated into a learning process to support SAGE data mining. Boratyn *et al*. [46] introduced a distance function, which makes use of the functional class information of annotated genes along with the experimental data, for clustering gene expression data. Based on the construction of a binary matrix that represents gene membership in a set of biological functions, they decreased the distance between a pair of genes annotated with similar functions and increased the pair-wise distance if two genes were annotated with different functions, or if these genes have no annotation. The new distance function was implemented within hierarchical clustering and evaluated using a human cancer SAGE dataset including 258 tags, whose expression values were significantly different across 4 normal and 7 ductal carcinoma *in situ *samples [47]. An improvement in terms of biological validity of the obtained clusters was observed when using the combined distance measure in comparison to results from traditional data-driven clustering. This study made a strong case for the inclusion of existing biological information for supporting clustering-based SAGE data analysis.

## Clustering evaluation techniques

As an unsupervised approach, clustering techniques generally do not require an external teacher to oversee a learning process and there are no predefined classes to indicate the type of valid relations or patterns that should be expected from the clustering process. Thus, the evaluation of clustering results is an essential task in cluster analysis.

Traditionally, the development of cluster validation techniques has mainly relied on indicators inferred from the data. One such example is the utilization of various cluster validity indices, which incorporate statistical aspects of the resulting partitioning to provide a quantitative assessment of the quality of clustering results. For example, based on the combination of different inter- and intra-cluster distances, Wang *et al*. [48] implemented generalized *Dunn's cluster validity index *to support clustering-based, large-scale analysis of SAGE expression data generated in the developing mouse retina.

It has been shown that such data-driven cluster evaluation methods are not sufficient for clustering analysis of biological data [30]. Given the fact that one of the most important objectives of clustering analysis of SAGE data is to identify biologically-meaningful expression patterns encoded in the data, recent years have seen a growing trend towards the incorporation of prior biological knowledge to assess the quality of the clustering outcomes. Using functional information available in the Gene Ontology database, for instance, Datta and colleague [48] proposed the following two measures for assessing biological relevance of clustering analysis of gene expression data, including a Human breast cancer SAGE data: (a) *Biological homogeneity index*, which assesses how biologically homogeneous the clusters are, and (b) *biological stability index*, which measures the consistency of biological results produced. In an attempt to support the generation of biologically-meaningful partitioning, Zheng *et al*. [29] applied the *hypergeometric distribution *test to quantitatively assess the level of functional class enrichment (or over-representation) in a given partition. For each class (e.g. biologically category, cancer-specific SAGE tags), the probability (*p*-value) of observing *k *tags belonging to a class within a given cluster by chance is computed using the following formulae:

(6) $$p=1-\sum_{i=0}^{k-1}\frac{\left(\begin{array}{c} K\\ i \end{array}\right)\left(\begin{array}{c} N-K\\ n-i \end{array}\right)}{\left(\begin{array}{c} N\\ n \end{array}\right)}$$

Where *K *is the number of tags assigned to the cluster under analysis, *k *is the number of tags belonging to the specific biological class in the cluster, *N *is the total number of SAGE tags in the whole data set and *n *is the total number of tags belonging to the specific class in the whole data set. If this probability is sufficiently low for a given class, one may conclude that the given biological category or functional class is significantly enriched in the cluster.

## Final remarks

Given the amount and complexity of the data, computational approaches play an essential role in the analysis of SAGE data. This paper concentrated on relevant clustering techniques for the SAGE domain, based on an assessment of their merits, disadvantages and applications. Although this paper did not intend to represent an exhaustive review, key techniques and application principles of clustering-based approaches to SAGE data mining were discussed. Emphasis has been placed on current advances in the development of clustering algorithms for knowledge discovery in SAGE data.

Clustering analysis of SAGE data has traditionally focused on the application of software and tools typically applied to microarray data analysis. However, there are some fundamental mathematical differences between SAGE and micro array studies [17,29]. For example, it has been shown that SAGE data are governed by different statistical models from those describing microarray data [17,44]. Recent studies have demonstrated that without considering such a distinguishing statistical nature, clustering-based SAGE data mining may fail to produce biologically-meaningful and statistically-valid results. New clustering algorithms, which model the Poisson statistical nature of SAGE data, were reviewed in this paper. The advantages in terms of their ability to improve SAGE pattern discovery and visualization were highlighted.

It is worth noting that the Poisson-based clustering techniques discussed in this paper are not without their own limitations. As Kim et al. [49] pointed out, PoissonC fails to take the direction of departure of observed from expected into account. Thus, they proposed a new distant measure, which emphasizes the profile shape through suitable data transformations. In addition, PoissonC [17], Poisson S and PoissonHC [43] are also limited by some of the factors exhibited by traditional clustering. For example, like the standard SOM-algorithm, the network topology needs to be specified by the user. Like *k*-means algorithms, PoissonC may be trapped in a local optimal depending on the selection of initial cluster seeds. Incorporation of Poisson statistics into alternative, advanced clustering techniques deserves further investigation.

Recent development in SAGE research has proposed some new mathematical models to analyze SAGE data [50-52]. For example, Zuyderduyn [50] proposed a Poisson mixture model to represent SAGE data. Gilchrist *et al*. [51] introduced a Bayesian framework to model SAGE tag formation and its effects on data interpretation. Integration of these models into SAGE clustering analysis would be part of the future work.

There is no universal clustering solution for SAGE data analysis and no single clustering technique can always perform well on different type of datasets. Therefore, in practice, it is recommended to use more than one clustering technique in order to achieve more reliable clustering results. The application of a hybrid approach such as the neuro-hierachical approach proposed by Wang et al. [27] also represents a promising way for large-scale clustering analysis of SAGE data. Such a combination, on the one hand, can reduce the size of the dimensionality of the input SAGE data and provide a user-friendly visualization platform to understand the overall structure of the data by allowing the user to inspect the resulting PoissonS map. On the other hand, by visualizing the resulting map produced by PoissonS and the dendrogram generated by PoissonHC, inter- and intra- cluster relationships encoded in the SAGE data may be readily detected and easily understood.

As a final step of clustering analysis, the application of cluster validation techniques is vital to assist users in understanding some fundamental questions such as: Are these clusters biologically meaningful? Does this cluster represent outliers or some novel findings? Both data- and knowledge- driven cluster validation techniques were introduced in the paper. It should be emphasized that, a vast collection of cluster assessment methodologies developed for microarray studies such as Gene Ontology-based cluster validation [53] can, in principle, be applied to SAGE data analysis. Finally, it is important to recognize that, in order to obtain statistically-reliable and biologically-meaningful results, the application of both internal and external validation techniques is recommended for the assessment of clustering outcomes [30].

## Competing interests

The authors declare that they have no competing interests.

## Authors' contributions

HW, HZ and FA co-wrote the manuscript. All authors read and approved the final manuscript.
